# Association between BMI Change, Transaminases, and Other Metabolic Parameters in Children with Nonalcoholic Fatty Liver Disease

**DOI:** 10.1155/2024/6997280

**Published:** 2024-05-23

**Authors:** Alvaro G. Flores Lopez, Ruben E. Quiros-Tejeira, Elizabeth Lyden, Brooke McGill, Chinenye R. Dike

**Affiliations:** ^1^Department of Pediatrics, Division of Pediatric Gastroenterology, Hepatology and Nutrition, University of Nebraska Medical Center, Children's Nebraska, Omaha, NE, USA; ^2^Department of Biostatistics, University of Nebraska Medical Center, Omaha, NE, USA; ^3^Creighton University School of Medicine, Omaha, NE, USA

## Abstract

**Background:**

Weight loss and lifestyle interventions are the mainstay of treatment in pediatric NAFLD. There are gaps in the literature on the objective improvement in BMI to meaningfully impact NAFLD in children.

**Aim:**

To determine the decrease in BMI associated with a significant decline in ALT and other metabolic parameters.

**Methods:**

Retrospective chart review of pediatric patients with the diagnosis of NAFLD. Data were collected at the baseline and 6 and 12 months. A linear regression model was used to assess the percent change in BMI predictive of change in ALT and other metabolic parameters.

**Results:**

281 charts were included. 71% of patients who had up to a 2.5% loss in BMI at 6 months had a decrease in ALT of up to 10 U/L compared to 43% patients who did not have a decrease in BMI up to 2.5% loss at the same time period (*P*=0.01). The linear regression model showed that 6-month and 12-month percent changes in BMI are predictive of 6-month and 12-month ALT changes (*P*=0.01 and 0.02), respectively. ALT normalization was achieved on 12% of patients with a ≥2.5% decrease in BMI at 6 months compared to 1% of patients that had no decrease of ≥2.5% decrease in BMI at 6 months (*P*=0.01). The mean BMI *Z*-score decline was 0.18 (*P*=0.001) in the group with a ≥2.5% decrease in BMI at 6 months.

**Conclusions:**

BMI loss of up to 2.5% and the mean BMI Z-score 0.18 are associated with a significant decrease in ALT of up to 10 U/L. BMI percent change at 6 months and 12 months is predictive of changes in ALT. These results should help guide providers in clinical practice set objective goals for the management of children with NAFLD resulting from obesity.

## 1. Introduction

Nonalcoholic fatty liver disease (NAFLD) is the most common cause of chronic liver disease in children and adolescents in the United States [1]. The rise in the prevalence of obesity likely explains the emergence of NAFLD as the leading cause of liver disease in children worldwide [2]. The prevalence of NAFLD in children is currently estimated to be 5–10% of the general population and has become the leading indication for liver transplantation in young adults (<50 years of age) in the United States, although transplant in children remains a rare occurrence [3].

NAFLD in children is on a spectrum of liver abnormalities secondary to fat accumulation in the absence of other etiologies including storage metabolic conditions, infections, autoimmune processes or hepatotoxic medications, and toxins [3, 4]. Phenotypic variations in nonalcoholic fatty liver diseases include steatosis and hepatocellular inflammation that might progress to fibrosis and cirrhosis [1, 3, 4].

Most children with NAFLD and nonalcoholic steatohepatitis (NASH) have obesity [5]. The prevalence of NAFLD in patients with obesity is estimated to be 26% and up to 59% in patients' severe obesity [3]. The presence of a cardiometabolic risk factor such as insulin resistance, prediabetes, diabetes, dyslipidemia, or central adiposity in an overweight child also increases the risk of NAFLD. NAFLD is more common in Hispanics [6]. Other risks for NAFLD include genetic predisposition, obstructive sleep apnea, and panhypopituitarism [1]. NAFLD in children seems to be primarily polygenic, and several genes (e.g., PNPLA3, TM6SF2, and PARVB) have been associated to the severity of steatosis and fibrosis in children with NAFLD. The majority of children with the most common mutation (PNPLA3) associated to NAFLD were Hispanics which suggest genetic racial/ethnic links [7]. Risk factors such as race/ethnicity (Hispanics and Asians), sex (males), socioeconomical status (middle to high income populations), and changes in obesity trends seem to affect the distribution of NAFLD, and certain geographical areas have shown increased rates of NAFLD (Latin America, North America, north Africa, and Middle East); however, irrespective of sex and geographic location, NAFLD prevalence in children and adolescents is increasing worldwide [8].

Current management of NAFLD in children is focused on weight loss and lifestyle modifications [1]. Weight loss has been shown to reverse NAFLD and NASH, particularly if initiated early in the course of the disease before advanced fibrosis has developed [1]. Counselling on lifestyle modifications to achieve weight loss in children is often unsuccessful given associated psychological comorbidities [9].

It is known that a decrease of 10 U/L in alanine aminotransferase (ALT) in children is associated with 1.37 relative odds of resolution of NASH at 96 weeks [1, 10, 11]. A weight loss of 10% or greater and a decrease in BMI of greater than 5% from the baseline have been shown to improve ALT in adults [12]. However, there are limited data in children on the association of BMI decline and ALT (5, 13–16). Given the difficulty associated with weight loss in adolescents, it is important to investigate the amount of BMI percent and BMI *z*-score decrease in children that would lead to an ALT decline of 10 U/L or greater and subsequently lead to NASH resolution.

The aim of this study is to determine the amount of BMI percent and BMI *Z*-score decline in children with NAFLD associated with up to a 10 U/L decrease in ALT.

## 2. Methods

This is a retrospective cohort study of children 0–19 years followed at the Children's Hospital and Medical Center Omaha from Dec. 2009 to Dec. 2019 with a diagnosis of NAFLD and NASH by ICD9 diagnosis. We collected clinical information including, systolic/diastolic blood pressure, baseline comorbidities (obstructive sleep apnea, systemic hypertension, diabetes mellitus, polycystic ovary syndrome, dyslipidemia, metabolic syndrome, hypothyroidism), and demographic information (age, gender, race/ethnicity). We also collected data on anthropometric variables (BMI, BMI *z*-score, and weight) transaminases (Alanine aminotransferase or ALT, aspartate aminotransferase or AST, gamma-glutamyl transferase or GGT) at the first visit or within 1 month of first visit, at 6 months, and at 12 months. We used data from within 2 months (before or after) of the 6 months and 12 months visits if transaminases were unavailable at 6 and 12 months. Data on other metabolic variables including HbA1c (hemoglobin A1c), insulin level, LDL (low-density lipoprotein cholesterol), HDL (high-density lipoprotein cholesterol), total cholesterol, triglyceride levels, and fasting blood glucose were collected at the baseline and 6 and 12 months. Data were entered into REDCap.

Our analysis included only patients who met the inclusion criteria of the abnormal ALT level per NAFLD North American Society for Pediatric Gastroenterology, Hepatology, and Nutrition (NASPGHAN) guidelines (ALT ≥26 U/L in males and ≥22 U/L in females). We then compared BMI percentile decline (≥5% and ≥2.5%) and BMI *z*-score (≥0.25) with decrease of ALT (≥10 U/L and ≥5 U/L) to determine associations between BMI decline and ALT values at 6 and 12 months. ALT normalization was defined as normal ALT values per NASPGHAN NAFLD guidelines: <26 U/L in males and <22 U/L in females at 6 and 12 months.

We compared clinical (blood pressure (systolic and diastolic), baseline comorbidities, clinic visit) demographic, anthropometric (BMI, BMI *Z* score and weight), and metabolic parameters (HbA1c, GGT, AST, insulin level, LDL, HDL total cholesterol, triglyceride levels, fasting blood glucose) at the baseline in the group of patients who had up to a 2.5% BMI percentile decline versus those who did not. Descriptive statistics were used to summarize demographics and clinical characteristics of the patients. Fisher's exact test was used to compare categorical variables between the groups, while continuous data were compared between the groups using the *t*-test or the Mann–Whitney test. A linear regression model was used to assess if a 6-month percent change in BMI was predictive of 6-month change in ALT, HbA1c, HDL, LDL, total cholesterol, and triglycerides. Model assumptions were verified using diagnostic plots. Detailed results of the regression models with diagnostic plots are shown in Appendix 1 and Appendix 2. Analysis was done using SAS, Version 9.4, and a *P* value of <0.05 was considered statistically significant.

## 3. Results

**Figure 1:**
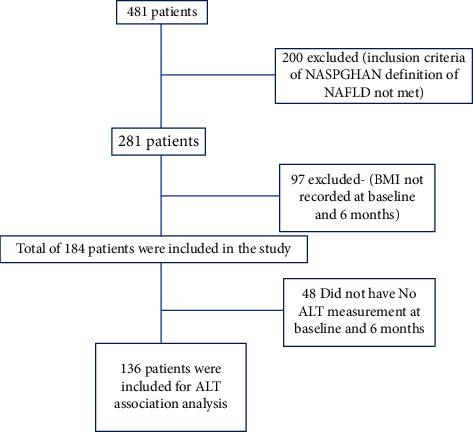
Flow chart of patients included in the study.

481 charts were reviewed (see flow chart in Figure 1). Most of the analyzed cohorts were males 61%, Hispanics 57%, attended a weight management program (52%), and had a mean age of 12 years old. Other demographic information is found in Table 1. 55% had dyslipidemia, 22% had obstructive sleep apnea, 11% had diabetes, 11% had hypertension, 10% metabolic syndrome, 4% hypothyroidism, and 5% had polycystic ovary syndrome. None of the patients were taking hepatotoxic or weight loss medications, and also none had concurrent diagnosis of liver diseases (e.g., alpha-1 antitrypsin deficiency, autoimmune liver disease, viral hepatitis (B and C), hemochromatosis, and Wilson's disease), history of weight loss surgery, or liver transplantation.

There were no significant differences between the groups (≥2.5% BMI loss versus no BMI loss of ≥2.5%) in baseline demographics, baseline transaminases (AST, ALT, GGT), anthropometrics (BMI, BMI *z* score, weight), baseline comorbidities (obstructive sleep apnea, systemic hypertension, diabetes mellitus, polycystic ovary syndrome, dyslipidemia, metabolic syndrome, hypothyroidism) and other metabolic variables (HbA1c, GGT, AST, insulin level, LDL, HDL total cholesterol, triglyceride levels, fasting blood glucose), and systolic/diastolic blood pressure (Table 1), and there was no difference between patients who attended a weight management program.

**Figure 2:**
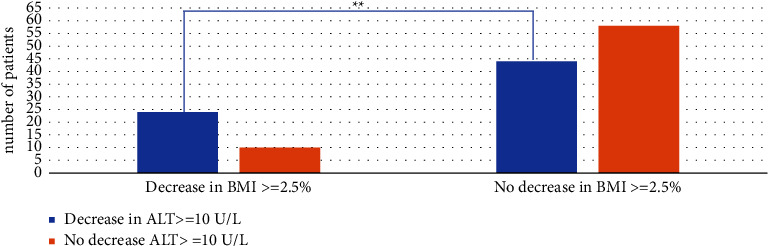
Number of patients who had a reduction of ≥2.5% BMI and decrease in ALT ≥10 U/L compared to patients who did not have BMI reduction ≥2.5% at 6 months.

71% of patients who had up to a 2.5% decline in BMI at 6 months had a decrease in ALT of up to 10 U/L compared to 43% patients who did not have a decrease in BMI of up to 2.5% at the same period (*P*=0.01) (Figure 2). 79% of patients who had a decrease of up to 2.5% in BMI at 6 months had a decrease in ALT of up to 5vU/L compared to 51% of patients who did not have up to 2.5% decrease in BMI at 6 months (*P*=0.01). There were no significant associations between ALT decline in patients who had a decrease of up to ≥0.25 BMI *Z* score at 6 months; however, statistical significance was achieved for BMI *Z*-score with mean loss 0.18 (*P*=0.01) in the group of >2.5% BMI decline at 6 months. ALT normalization was achieved on 12% of patients with a ≥2.5% decrease in BMI at 6 months compared to 1% of patients that had no decrease of ≥2.5% decrease in BMI at 6 months (*P*=0.01).

**Figure 3:**
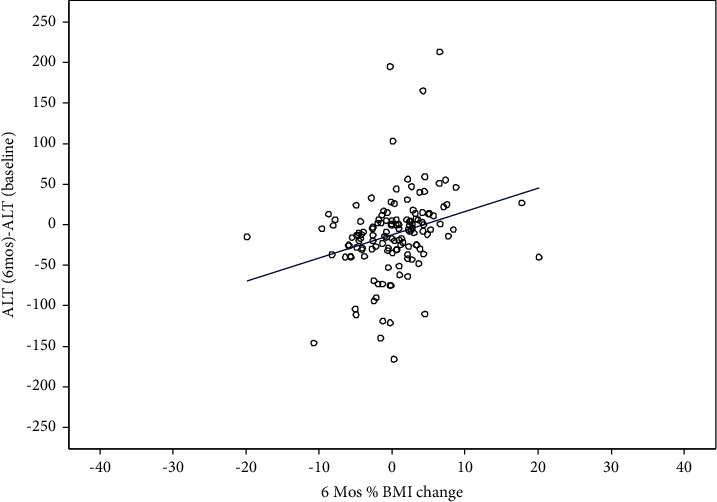
Plot of 6-month BMI and ALT change from the baseline.

**Figure 4:**
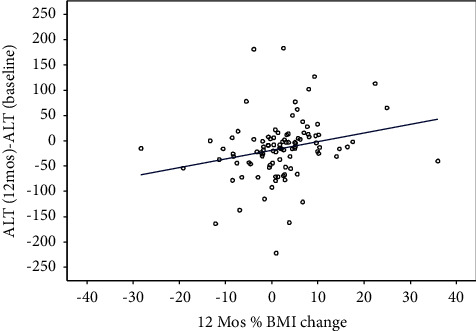
Plot of 12-month BMI and ALT change from the baseline.

The linear regression model showed that a 6-month (*N* = 136) and 12-month (*N* = 99) percent change in BMI is predictive of 6-and 12-month ALT changes (*P*=0.01 and 0.01), respectively. For every 1% increase in BMI over the 6-month and 12-month periods, there is an expected increase in ALT of 2.9 U/L and 1.7 U/L, respectively (Figures 3 and 4). There were no significant associations between 6-month percent BMI changes with total cholesterol, HDL, LDL, Hb A1c, and triglycerides. Similarly, a 2.5% decline in BMI percent was not associated with significant changes in these metabolic parameters, but there was an improvement in these parameters in patients with a 2.5% decline in BMI percent when compared to those who did not have this decrease (Table 2).

## 4. Discussion

Our results show that a decrease in BMI percent of up to 2.5% at 6 months is significantly associated with a decrease in ALT of up to 10 U/L in children with NAFLD. Similarly, our model showed that a 6- and 12-month percent change in BMI is associated with an ALT change. ALT normalization was achieved in 12% of patients with a ≥2.5% decrease in BMI percent at 6 months compared to 1% of patients that had no decrease of ≥2.5% in BMI at 6 months (*P*=0.01). For every 1% increase in BMI percent, there is an associated ALT change of 2.9 U/L and 1.7 U/L at 6 and 12 months, respectively.

In the COVID era, obesity incidence has increased to epidemic levels and the secondary rise in NAFLD will pose new challenges to healthcare systems and pediatric providers including the pediatric gastroenterologist and transplant hepatologist [13]. Focus on prevention and management of patients with NAFLD should be a priority to stall the advancement of the disease and its complications.

Besides lifestyle modifications, most treatment modalities have proven to be unsuccessful for treatment of NAFLD in children [14], but lifestyle modifications leading to weight loss are difficult to implement in children particularly adolescents [9]. Current NASPGHAN guidelines recommend lifestyle modifications as the first-line intervention for the management of NAFLD and the use of ALT decrease or normalization as an acceptable surrogate in NAFLD treatment. These guidelines have also suggested specific values of normal ALT by sex (<26 U/L in males and <22 U/L in females at 6 and 12 months) and developed an algorithm of diagnosis and management for NAFLD in children based on ALT changes (ALT cutoffs have been validated from US nationally representative cohorts) [1].

Studies that evaluate weight loss and improvement on BMI have been performed in children and adults and have demonstrated improvement in metabolic outcomes with specific interventions. A decrease in the BMI >5% and/or weight loss >10% has been found to lead to reductions in waist circumference, cholesterol, triglycerides, and improvement in insulin resistance parameters in children and adults, [6, 7]. Despite the evidence on the impact of weight loss in fatty liver disease and other variables in children, there are still gaps on the impact on BMI improvement in ALT values [4].

In 2013, St-Jules et al. studied the effects of BMI *z*-score and ALT improvement in Hawaiian children and did not find an association between BMI reduction and ALT improvement despite overall improvements in transaminases with the interventions. They concluded that body weight is not an appropriate primary outcome in obese pediatric NAFLD patients and greater emphasis should be placed on patient adherence with modifiable behaviors [15]. In contrast, our study showed a significant association between BMI decrease and ALT improvement (Figure 1). It is possible that our bigger sample size and the race/ethnicity of our cohort accounted for this difference in outcomes. Flegal et al. also utilized BMI *Z*-score as their primary outcome which has been found to be less reliable for extreme values [16]. Although our results did not show any significant association between BMI *z*-score and ALT levels with 0.25 BMI *Z*-score decline, (number chosen based on the results of previous studies) the patients that had a decline of at least 2.5% BMI had a mean BMI *Z*-score decline of 0.18. We hypothesized that these results might be secondary due to the effect of not enough power since the number of patients that reach the 0.25 BMI *Z*-score decline outcome was small.

The POWER study published in 2019 identified that a decrease of 5.2% in BMI% 95^th^ percentile was associated with the normalization of an initially abnormal ALT at 6 months [17]. Our study showed that a 2.5% decrease in BMI was associated with an improvement in ALT levels by at least 10 U/L. This difference in results may be because we utilized more stringent criteria from the NASPGHAN NAFLD guidelines cut off for ALT (ALT ≥26 U/L in males and ≥22 U/L in females), while their cut off was ALT ≥40 U/L in both males and females. Also, we utilized BMI percent change rather than percentile which might represent higher percentile loss.

Utz-Melere et al. [18] in 2018 performed a systematic review and meta-analysis regarding dietary and physical activity interventions in 2018, which included 19 articles. They concluded that lifestyle changes lead to significant improvements in BMI. Some of the authors tried to estimate the amount weight loss needed for a significant improvement of NAFLD and found that results ranged from 1 to 10% weight loss to obtain some benefit. Outcomes used in this study were heterogenous. The largest study that addressed the impact of BMI decline on transaminases was that by Reinehr et al. [19]. They performed a 1-year multidisciplinary lifestyle intervention on 160 German children (109 received the intervention) and indicated that transaminases normalize even with very minimal improvement in BMI *Z*-score (>0–0.25). They overall saw a mean improvement of 11 U/L at the end of the intervention, but they only utilized BMI *Z*-score in their outcomes.

Many of our patients demonstrated weight gain, and we assessed the effect of weight gain on the ALT with a linear regression model and found that for every 1% increase in BMI at 6 months, the increase in ALT will be 2.9. (*P*=0.01) and 1.7 at 12 months (*P*=0.02). Similarly, the power study demonstrated that a 0.48% BMIp95 increase in BMI was associated with an abnormal ALT at 6 months from a normal value at the baseline [17]. The power study found an association between a decrease in BMI with improvement in other metabolic variables. In our study, patients who had ≥2.5% BMI decline demonstrated improved trends in all metabolic variables (Table 2), but these improvements were not significant. This may be because of our smaller sample size.

We used the NASPGHAN NAFLD guidelines for cut off for normal ALT which represents a major strength our study. This lower cut off provides a higher sensitivity to capture more children with NAFLD and tighter associations with BMI trends. The retrospective nature of the study including missing information related to possible cofounder (diet, physical activity, etc.), analysis of only about 28% of the cohort, and absence of biopsy proven NAFLD diagnoses are some of the limitations of this study. There are limitations for using ALT for the assessment and management of pediatric NAFLD, but ALT is still widely used because it is cost-effective and noninvasive compared to other methods such as liver biopsy and MRI due to the need of sedation and increased radiation exposure with CT scan. Conventional ultrasound is not accurate enough for the assessment of NAFLD in children due to interobserver variability, and newer noninvasive methods such as elastography are not yet widely available, and there are still limitations in the pediatric population [20].

## 5. Conclusions

Our study showed that BMI decline of up to 2.5% and mean BMI *Z*-score decline of 0.18 are associated with a significant decrease in ALT of up to 10 U/L in children with obesity. ALT normalization was achieved in up to 12% of patients, and BMI percent changes at 6 months and 12 months were predictive of a change in ALT. These results provide understanding regarding the associations between BMI and weight changes and the impact on ALT in pediatric patients. This should help guide providers in clinical practice on the objective goals and metrics of success for the management of children with NAFLD resulting from obesity.

Larger prospective studies are needed to confirm these findings with the use of histopathology and other imaging modalities (MRI PDFF) to assess in detail the changes in steatosis and fibrosis in pediatric NAFLD. Although biochemical parameters such as ALT are widely used and inexpensive, due to its limitations, there is a strong need for other noninvasive modalities such as elastography to improve the management of pediatric NAFLD.

## Figures and Tables

**Table 1:** Baseline demographic comparison between patients that had ≥ 2.5% decrease in BMI and no decrease in BMI ≥ 2.5%.

Variables	BMI decrease ≥ 2.5%				No decrease in BMI ≥ 2.5%				*P* values
	*N*	Mean	Std dev	Median	*N*	Mean	Std dev	Median	
Age (years)	43	12.26	3.57	12.00	141	12.3	3.00	12.00	0.90
Weight (kg)	43	80.39	27.86	70.90	141	84.57	30.00	79.60	0.42
Weight *Z*-score	42	2.38	0.85	2.46	138	3.65	14.38	2.52	0.30
Height (cms)	43	158.06	18.75	159.69	141	156.33	14.11	157.61	0.58
BMI (kg/m^2^)	43	31.31	6.39	30.17	141	33.76	7.73	32.80	0.060
BMI *Z* score	42	2.16	0.61	2.30	138	2.29	0.63	2.41	0.26
Waist circumference (cms)	16	37.70	6.76	38.25	69	39.95	7.74	37.50	0.29
Hemoglobin A1C (%)	21	5.77	1.35	5.50	66	5.92	1.59	5.40	0.69
Systolic blood pressure (mmHg)	41	117.49	14.27	122.00	140	119.98	16.01	119.50	0.37
Diastolic blood pressure (mmHg)	41	66.54	9.55	66.00	140	71.05	9.89	70.50	0.010
LDL cholesterol (mg/dl)	22	97.65	23.22	94.50	76	106.58	31.20	107.50	0.22
HDL cholesterol (mg/dl)	22	35.95	8.03	33.50	76	37.80	8.37	38.00	0.36
Total cholesterol (mg/dl)	24	164.25	29.44	165.00	76	174.79	37.76	176.00	0.21
Triglycerides (mg/dl)	22	164.18	109.39	135.50	76	196.13	117.52	176.50	0.26
Blood glucose (mg/dl)	39	111.87	122.30	94.00	132	102.21	46.28	94.00	0.63
ALT (alanine aminotransferase) (U/L)	43	82.12	50.72	66.00	141	97.08	73.52	75.00	0.13
AST (aspartate aminotransferase) (U/L)	43	57.72	37.63	49.00	141	60.96	38.52	49.00	0.63
GGT (gamma glutamyl transferase) (U/L)	20	60.30	97.85	32.50	62	47.39	39.20	31.00	0.57

**Table 2:** 6 months change comparison between patients that had ≥ 2.5% decrease in BMI and no decrease in BMI ≥ 2.5%.

Variables	BMI decrease ≥ 2.5%				No decrease in BMI ≥ 2.5%				*P* values
	*N*	Mean	Std dev	Median	*N*	Mean	Std dev	Median	
Weight (kg)	43	2.59	3.93	−2.20	141	3.48	3.52	2.99	0.01
Weight *Z*-score	40	−0.23	0.17	−0.22	137	0.06	21.06	−0.02	0.30
Height (cms)	39	2.75	2.87	2.31	136	2.33	2.15	2.60	0.01
BMI *Z* score	40	−0.18	0.19	−0.18	136	0.02	0.18	−0.01	0.01
Waist circumference (cms)	13	−1.47	1.72	−1.50	47	0.54	1.75	0.50	0.01
Hemoglobin A1C (%)	8	−0.06	0.36	−0.05	32	0.06	0.68	0.05	0.72
LDL cholesterol (mg/dl)	8	−13.13	23.04	−10.50	39	−6.06	21.50	−5.00	0.40
HDL cholesterol (mg/dl)	8	2.25	6.92	5.00	39	1.67	6.27	1.00	0.69
Total cholesterol (mg/dl)	9	−20.33	31.48	−12.00	39	−7.33	21.99	−13.00	0.41
Triglycerides (mg/dl)	8	−39.00	67.24	−16.50	39	−34.18	120.43	−16.00	0.49
Systolic blood pressure (mmHg)	37	3.95	15.70	6.00	133	1.19	19.07	0.00	0.33
Diastolic blood pressure (mmHg)	37	0.03	12.04	2.00	133	−1.69	13.23	−1.00	0.47
AST (aspartate aminotransferase) (U/L)	34	−15.65	36.68	−10.50	101	−3.25	47.87	−7.00	0.25
GGT (gamma glutamyl transferase) (U/L)	13	−7.08	15.28	−2.00	41	5.37	29.72	0.00	0.16
Blood glucose (mg/dl)	28	0.71	11.83	−1.00	92	2.43	21.56	0.00	0.96

## Data Availability

The statistical data and bibliographic references used to support the findings of this study are included in the article.

## Supplementary Materials

Supplementary Materials Appendix 1: Regression model of 6 month percent change in BMI predicting 6 month change in ALT. Appendix 2: Regression model of 12 month percent change in BMI predicting 12 month change in ALT.
