# Supplementary material for: Association between BMI Change, Transaminases, and Other Metabolic Parameters in Children with Nonalcoholic Fatty Liver Disease
Source: J Obes. 2024 May 23;2024:6997280. doi: 10.1155/2024/6997280 (PMC11139528; doi:10.1155/2024/6997280)
Supplement: Supplementary Materials — Appendix 1: Regression model of 6 month percent change in BMI predicting 6 month change in ALT. Appendix 2: Regression model of 12 month percent change in BMI predicting 12 month change in ALT. [file 6997280.f1.zip › Appendix. 1.docx]

**Appendix 1**

**Regression model of 6 month percent change in BMI predicting 6 month change in ALT**

Assumptions of Regression Model:

For normality, linearity and homoscedasticity, please refer to the residual plots. There is some departure from normality as evidenced in the second plot in the left column. Linearity and homoscedasticity assumptions appear to be met based on the first plot (residuals and predicted values) in the left column.

Note: The Durbin-Watson test was not performed because we are not dealing with time series data. For each patient, 6 month percent change in BMI was determined by subtracting baseline BMI from the 6 month BMI value and dividing by baseline BMI. For each patient, 6 month change in ALT was determined by subtracting the baseline ATL from the ALT recorded at 6 months.

Statistical significance, model fit and coefficient of determination for the Regression Model:

F-statistic: 10.70

df: 135

p-value: 0.0014

R-Square: 0.0739

Adj R-Sq: 0.0670

Root MSE: 49.879

Regression equation: Expected ALT 6 month change=-12.412+2.881*(BMI 6 Month Percent Change)

Sample size for model was 136 out of the 281 pediatric patients included the manuscript.
