# Supplementary material for: Association between BMI Change, Transaminases, and Other Metabolic Parameters in Children with Nonalcoholic Fatty Liver Disease
Source: J Obes. 2024 May 23;2024:6997280. doi: 10.1155/2024/6997280 (PMC11139528; doi:10.1155/2024/6997280)
Supplement: Supplementary Materials — Appendix 1: Regression model of 6 month percent change in BMI predicting 6 month change in ALT. Appendix 2: Regression model of 12 month percent change in BMI predicting 12 month change in ALT. [file 6997280.f1.zip › Appendix. 2.docx]

**Appendix. 2**

**Regression model of 12 month percent change in BMI predicting 12 month change in ALT**

Assumptions of Regression Model:

For normality, linearity, and homoscedasticity, please refer to the first two plots in the left column. Based on the plots, the assumption of normality, linearity and homoscedasticity appear to be met

Note: The Durbin-Watson test was not performed because we are not dealing with time series data. For each patient, 12 month percent change in BMI was determined by subtracting baseline BMI from the 12 month BMI value and dividing by baseline BMI. For each patient, 12 month change in ALT was determined by subtracting the baseline ATL from the ALT recorded at 12 months.

Statistical significance, model fit and coefficient of determination for the Regression Model:

F-statistic: 5.96

Corrected total df: 98

p-value: 0.0165

R-Square: 0.0579

Adj R-Sq: 0.0482

Root MSE: 58.892

Regression equation: Expected ALT 12 month change=-18.979+1.713*(BMI 12 Month Percent Change)

Sample size for model was 99 out of the 281 pediatric patients included the manuscript
